# Applying Nutrient Profiling Systems to Packaged Foods and Drinks Sold in Jamaica

**DOI:** 10.3390/foods9010065

**Published:** 2020-01-08

**Authors:** Suzanne Soares-Wynter, Stacey-Ann Aiken-Hemming, Bridget Hollingsworth, Donna R. Miles, Shu Wen Ng

**Affiliations:** 1 Caribbean Institute for Health Research, The University of the West Indies Mona, Kingston 7, Jamaica; suzanne.soareswynter@uwimona.edu.jm (S.S.-W.); staceyann.aiken02@uwimona.edu.jm (S.-A.A.-H.); 2 Carolina Population Center, The University of North Carolina at Chapel Hill, Chapel Hill, NC 27516, USA; bhollin@email.unc.edu (B.H.); drmiles@email.unc.edu (D.R.M.); 3 Department of Nutrition, The University of North Carolina at Chapel Hill, Chapel Hill, NC 27516, USA

**Keywords:** nutrient profiling system, food supply, packaged foods, beverages, Jamaica

## Abstract

The Pan American Health Organization (PAHO) and Chile stage III nutrient profiling systems (NPSs) were applied to packaged beverages/foods sold in Jamaica to: (a) identify products with excess nutrients of concern (NOC) under each NPS; (b) assess differences between these NPS, and (c) discuss the advantages and disadvantages of each NPS when applied to Jamaica’s food supply. Data on 6261 branded multi-ingredient packaged products were collected from the city of Kingston in 2018; of these, 4739 products, comprised of 3423 foods (from 15 food groups) and 1316 beverages (from four beverage groups), provided sufficient information. Products containing excessive NOC under each NPS were identified and the proportions of categories with excess NOC were compared using correlation coefficients. Also assessed were the mean nutrient values among the subset of products exceeding versus not exceeding both NPSs using tests of significance. A larger proportion of packaged beverages and foods exceeds thresholds under PAHO versus Chile Stage III. Additionally, a larger percentage of foods, like fruits, vegetables, legumes, fish and seafood, would be considered as having excess sugar or sodium under PAHO versus Chile. This is the first study in the Caribbean that applies two existing NPSs to packaged products. The results can help to determine an appropriate NPS for use in Jamaica as the basis for food and nutrition policies, to help consumers make improved food choices.

## 1. Introduction

Poor diets remain a leading cause of obesity and chronic non-communicable diseases globally [1]. Like many low- and middle-income countries, Jamaica is experiencing a rapid nutrition transition resulting in high rates of weight gain [2], and a consistent pattern of increasing obesity and diabetes prevalence among adults and children [3,4,5]. Preliminary results of the most recent national survey estimate 54% of Jamaican adults are now overweight or obese. Although obesity and lifestyle related non-communicable diseases have taken precedence, Jamaica retains pockets of childhood malnutrition, although they are not as severe (or prevalent) as in former decades. Empirical evidence shows unhealthy dietary risk factors, such as insufficient fruits and vegetables, and excess sodium, saturated fats and ultra-processed foods [6,7,8]. In response, Jamaica and other neighbors of the Caribbean Community (CARICOM—a regional and political consortium of Caribbean island States including Jamaica), have mandated a multi-sectoral, multi-level approach to combatting the obesity epidemic. Protecting children’s right to safe and nutritious food remains a top policy priority and efforts are already underway to restrict the sale and provision of unhealthy foods, especially in and around schools [9,10,11]. To facilitate the broader public health agenda of improving the Jamaican food landscape, a key action item involves updating food labelling systems and identifying which foods (or food groups) contain excess nutrients of concern (NOC). The aim is to secure mandatory, comprehensive and accurate labelling.

Nutrient profiling systems may be useful as they employ a method of classifying beverages and foods by the amount of nutrients each contains and apply thresholds that are aligned with a country’s desired diet and nutrition goals. The selected NOC are usually associated with the diseases having the greatest public health impact. Conceptually, a nutrient profiling system (NPS) assigns a ranking or designation of healthfulness or unhealthfulness to foods and may be used in a variety of applications including front-of-package (FOP) labels, unhealthy beverage or food taxes, and marketing restrictions [12]. However, the process of developing and validating a country-specific NPS can be complicated, and replicating or adjusting an existing model is a cost effective alternative for countries with limited resources.

No formal assessment of the nutrient content or thresholds of Jamaican foods has been reported in the published literature. In 2016, the WHO Regional Office for the Americas/Pan American Health Organization (PAHO) developed an NPS to assist its Member States (including Jamaica and the CARICOM) with differentiating between unhealthy processed beverages/foods versus healthier, unprocessed versions [13]. The PAHO NPS defines thresholds for six nutrients and places foods into categories (culinary ingredients, freshly prepared dishes, unprocessed or minimally processed foods, processed foods, and ultra-processed foods). These are distinguishable by the nature and origin of the base food constituent as well as the type and amount of ingredients added during processing. However, only two categories of food are used with the PAHO NPS: processed and ultra-processed.

As a prominent authority on health in Jamaica, the PAHO model is expected to influence labelling policies. However, to provide an objective review and performance assessment of the PAHO NPS on Jamaican foods, a primary aim of this study is to explore other NPSs for comparison. Many Latin American countries have developed an NPS unique to their food landscape and are at various stages of development and implantation in the fight against obesity. The Chile NPS appears the most comprehensive and widely tested [14,15,16] and was thus selected for this study comparison.

The Chile NPS was first legislated in 2012, with revisions in 2016, and its most recent iteration (Chile Stage III) occurred in 2019 [17]. This makes it an attractive option as several food label laws have been implemented to curb the excessive consumption of sugary drinks and ultra-processed foods and FOP warning label laws were implemented as a firm response to the obesity epidemic. There are a few key differences between the two NPSs. In the Chile NPS, products are assessed based on added ingredients (specifically added sugar, saturated fat and sodium) and nutrient levels. Products with higher naturally occurring levels of a nutrient (e.g., sugar in dried fruit, but no added sugar ingredients) are not subject to the nutrient thresholds [18].

Whether a product exceeds energy density thresholds is only considered if a product contains added sugar or added saturated fat. Products are excluded from being evaluated against nutrient thresholds in PAHO if they are considered single-ingredient culinary ingredients following the NOVA system [19] or do not contain any added fats, added sugars, low-calorie (often-termed artificial and/or non-caloric) sweeteners and added sodium. The PAHO system assesses sugar, saturated fat and sodium in relation to the energy (in kilocalories) content of the product and does not include an energy density threshold. The PAHO system considers additional characteristics of products, including total fat, trans fat, and the presence of low-calorie sweeteners. Table 1 provides an overview and comparison of the characteristics of the PAHO and Chile NPS.

Labelling standards for pre-packaged goods in Jamaica currently require clear displays in English of company/brand contact information, the country of origin, details of shelf life, a food description, a declaration of contents, and an ingredients listing. An update and revision of labeling standards are underway, with the aim of including more detailed food and nutrient requirements. The process is being directed by the CARICOM Regional Organisation for Standards and Quality (CROSQ), with stakeholder input from each of the CARICOM Member States. Jamaica is contributing to the process through the Bureau of Standards Jamaica (BSJ), the country’s leading authority on food safety. By applying both the PAHO and Chile NPS to Jamaican foods, policymakers will be better informed when implementing more complex regulatory or educational mechanisms (for example, labels for nutrient facts, FOP warnings, and health claims).

Given the lack of information about pre-packaged beverage and food products sold in the Caribbean, this paper seeks to provide descriptive analyses on the products in Jamaica with excess NOC under each NPS and assesses what drives the differences found between these NPS. The advantages and disadvantages of each NPS in the Jamaican context are discussed to help inform the NPS and thresholds considered for policies in Jamaica and the Caribbean region.

## 2. Materials and Methods

### 2.1. Data Collection of Packaged Beverage and Food Products

Data for this study were retrieved from the labels of prepackaged beverages and foods available from two retail locations in the capital city of Kingston (the main city with the greatest population density in Jamaica). Both locations were large supermarket outlets, deemed to have a wide variety of local and imported products. Both were located in mixed socio-demographic suburban neighborhoods. The second location tended to stock products by various small food companies. All data were collected between January and March 2018 by a team of five fieldworkers and lead by a nutritionist. For the data capture, the team visited the retail outlet and each person walked along an aisle to capture photographs of all available branded packaged beverage and food items. A digital camera was used to capture images of all visible information available on the entire label. Only items identified as being multi-ingredient packaged foods were included in the study. Fresh foods (e.g., fruits, vegetables, raw meats) were excluded from these observations. The procedure was repeated until all items in the supermarket were captured.

Data collectors were trained prior to the study period. Ten days of in-office training was conducted by two trainers covering research protocols, practice with photo taking, and the use of the Research Electronic Data Capture (REDCap) software for data entry [20,21]. An additional five days were spent conducting supervised training inside the retail center.

A total of over 40,500 images were captured from 6261 products from the two locations. All product photographs were downloaded, coded, and stored on a secure sever for later retrieval. The information in each product photograph was entered into REDCap. The procedure involved copying all visible data in photographs of the product labels including product information (type of product, brand, and manufacturer), package size, nutrient facts panel (NFP) information, ingredient list, serving size, number of servings, and any preparation instructions. Each food entry was reviewed and revised by the project supervisor as a means of quality control to ensure accuracy and completeness. Periodic reliability testing was also performed on approximately 10% of the sample. This involved taking repeat photographs and having the data double entered in the database and checked for accuracy and repeatability. No prices or person-identifiable information were collected. All data were collected and entered by the end of June 2018.

### 2.2. Application of the PAHO and Chile Stage III Nutrient Profiling Systems

**Figure 1:**
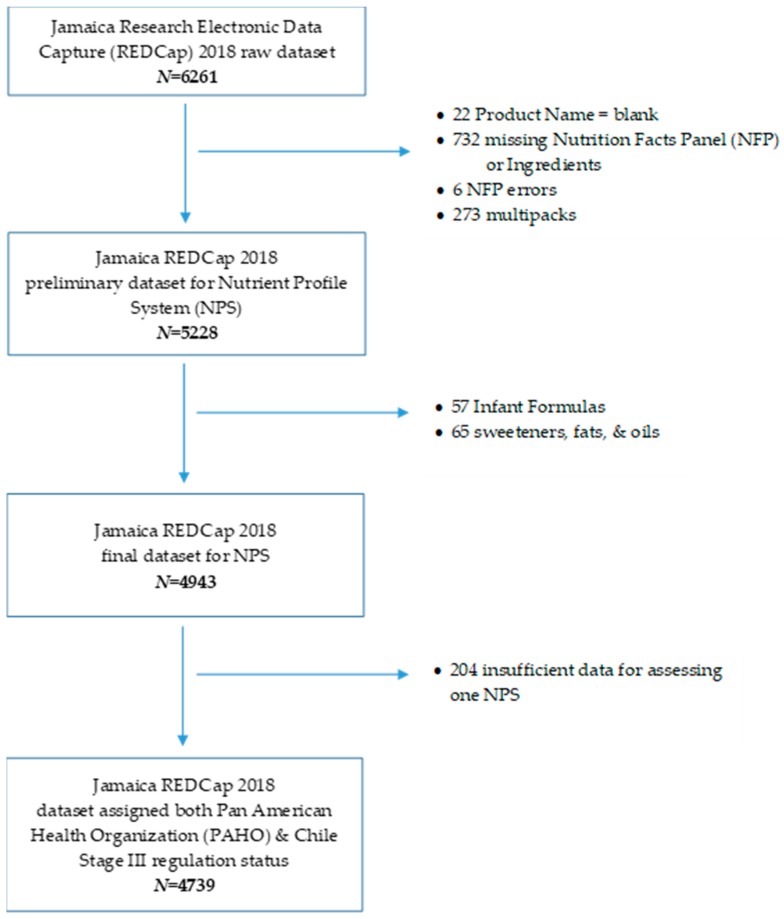
Exclusions from Data Sample. *N* = number of products.

As part of the data entry and review process, the 6261 products were originally categorized into one of 15 food categories and 18 beverage categories. Of these, 760 were excluded due to missing product name, NFP, ingredient list, or other errors identified in quality control measures. For ease of applying NPSs, 273 multi-pack products (items with multiple types of products such as variety packs of instant oatmeal) were excluded from this analysis. Next, 57 ‘Infant Formulas’ were excluded from this analysis, since these are not products typically consumed by the general population. As culinary ingredients are not subject to the NPSs, 65 sweeteners, fats, and oils were excluded; leaving 4943 products available for application of the two NPSs. After analysis and application of the NPSs, 204 products were excluded from the results of this paper due to insufficient data for assessing one of the systems. Consequently, the sample used for both NPSs was 4739 products, of which 3423 are foods and 1316 are beverages. A flowchart of exclusions from the data sample is shown in Figure 1.

There were 266 beverage products that required reconstitution with water, adding volume but not nutrients. Reconstitution factors were applied following manufacturers’ instructions for these products. Food products requiring preparation, typically adding nutrients (e.g., cake mixes that required addition of eggs, oil, water and baking), were not reconstituted for the purposes of this study.

From the resultant sample sizes in these categories, some categories were collapsed due to small sample sizes, resulting in 19 separate groups; four beverage groups, all in ready-to-drink forms: ‘100% Juice & Coconut Water’, ‘Dairy’, ‘Sodas & Flavored Beverages’, and ‘Other Beverages; and 15 food groups: ‘Bread & Bakery Products’, ‘Candy & Desserts’, ‘Cereal & Grain Products’, ‘Dairy Products’, ‘Dry Spices & Seasonings’, ‘Fats & Oils’, ‘Fish & Seafood’, ‘Fruits & Vegetables’, ‘Legumes’, ‘Meat & Eggs’, ‘Meat/Dairy Substitutes’, ‘Ready to Heat/Eat Foods’, ‘Sauces & Spreads’, ‘Snack Foods’, and ‘Sweeteners’.

The algorithms for Chile Stage III and PAHO NPSs were then applied to this sample to derive whether a product exceeded the threshold for the nutrients or characteristic of concern included in that particular NPS. A third NPS was also considered, termed the Alternate PAHO NPS, which only considers thresholds among the three nutrients of concern (NOC) that overlap in the Chile and PAHO NPSs (namely sugar, saturated fats and sodium). Figures S1 and S2 illustrate the algorithms used for the PAHO and Chile Stage III NPSs, respectively.

### 2.3. Descriptive Analysis

The share of products containing excessive amounts of NOC overall, among foods only, among beverages only, and by beverage/food group, were compared across the two NPSs. Differences in the proportion of “excess” NOC products under the PAHO NPS (total and alternate) and the Chile Stage III NPS were explored using tests of proportions and correlation coefficients. The difference in the proportion of products with two or more and four or more “excess NOC” between these models were also examined.

Next, two approaches were taken to understand why differences were noted between the two NPSs applied to Jamaican products. First, the proportion of each beverage and food category exceeding the NOC thresholds for the three nutrients covered in both NPSs were compared. Second, the mean nutrient values among the subset of products that overlapped in both NPSs, exceeded NOCs under PAHO but not under Chile, and did not exceed any NOCs in both NPSs, were compared. For all the analyses, *t*-tests were conducted and significance was established when *p <* 0.05. All statistical analyses were conducted using Stata version 15 (StataCorp, College Station, Texas, 2017) [22]. This secondary data analysis was deemed exempt from review by the University of North Carolina Institutional Review Board.

## 3. Results

### 3.1. Products with Excess Nutrients of Concern

**Figure 2:**
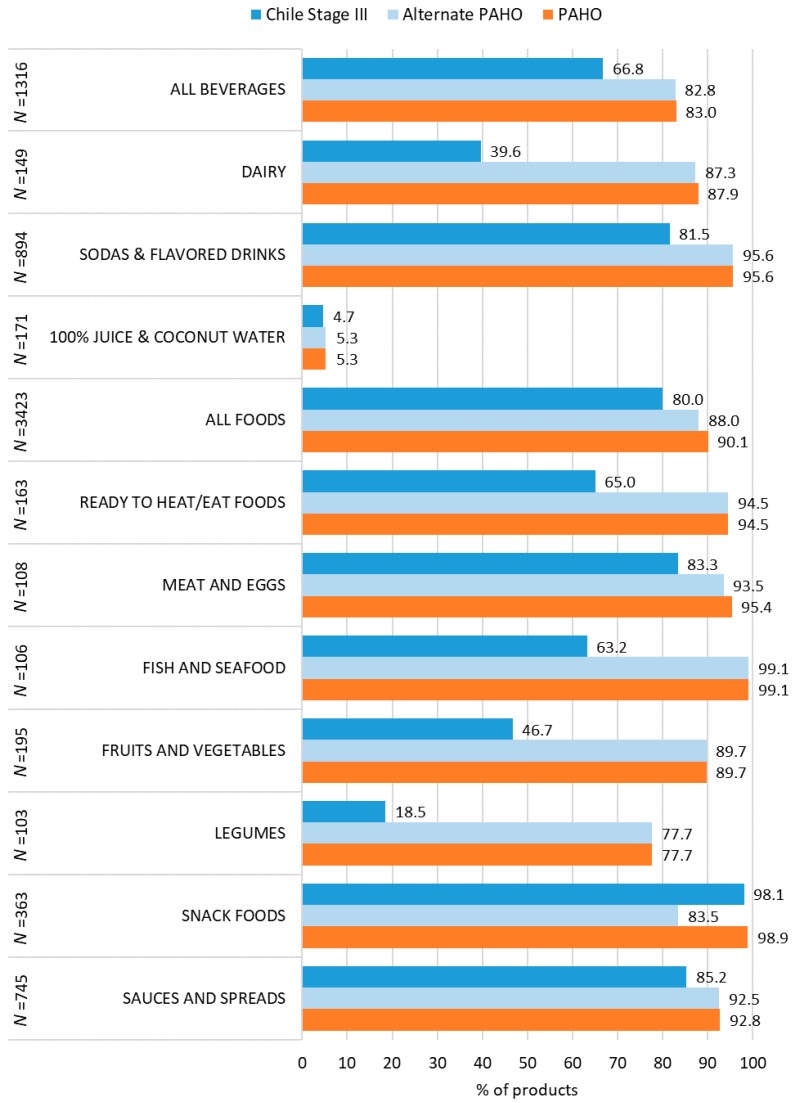
Proportion of beverage and food products with any “excess nutrients of concern” under the PAHO, Alternate PAHO and Chile Stage III nutrient profiling systems, by select groups. *N* = number of products; PAHO = Pan American Health Organization.

Figure 2 illustrates the proportion of products that exceed at least one of the thresholds under the two versions of PAHO and the Chile Stage III NPS across beverages, foods, and among select groups. In general, a larger percentage of beverage and food products exceed thresholds from the PAHO NPS compared to the Chile Stage III NPS. When applying the Alternate PAHO NPS, which only considers the nutrient thresholds of sugar, saturated fats and sodium, the share of products that exceed any NOC is very similar to when more nutrients or ingredients are considered under the PAHO NPS. The only exception was in the case of ‘snack foods’. As such, the results presented focus on the PAHO findings. Complete results for each of the beverage and food groups can be found in Tables S1 and S2.

Among beverages, the differences were significant between PAHO and Chile Stage III (83% vs. 66.8%, respectively, *p =* 0.00). The difference was particularly stark among ‘dairy drinks’, where 87% would exceed the PAHO thresholds, whereas only 39.6% of the dairy drink products would exceed the Chile Stage III thresholds (*p =* 0.00). Under both systems, a very high proportion of ‘sodas and flavored drinks’ would exceed thresholds, but this was still higher under PAHO than Chile Stage III (95.6% vs. 81.5%, respectively, *p =* 0.00). All of the reconstituted syrups were included in this group of ‘sodas and flavored drinks’, and, in sensitivity analyses excluding syrups, there was a negligibly lower proportion of products exceeding thresholds within this beverage group under both NPSs. Meanwhile, there was strong concurrence among ‘100% juice and coconut waters’ across the NPSs.

Among foods, 88%–90.1% would exceed the PAHO thresholds, whereas 80% would exceed the Chile Stage III thresholds (*p =* 0.00). The greatest differences were found for ‘fruits and vegetables’, ‘legumes’, and ‘fish and seafood’, as well as ‘ready-to heat/eat foods’. There was better alignment for ‘sauces and spreads’ where between 85%–93% of sauces and spreads exceeded at least one nutrient threshold. As noted previously, ‘snack foods’ was the only food group where the alternate PAHO NPS resulted in a lower percentage of products having any excess NOC, even compared to the Chile Stage III NPS.

**Figure 3:**
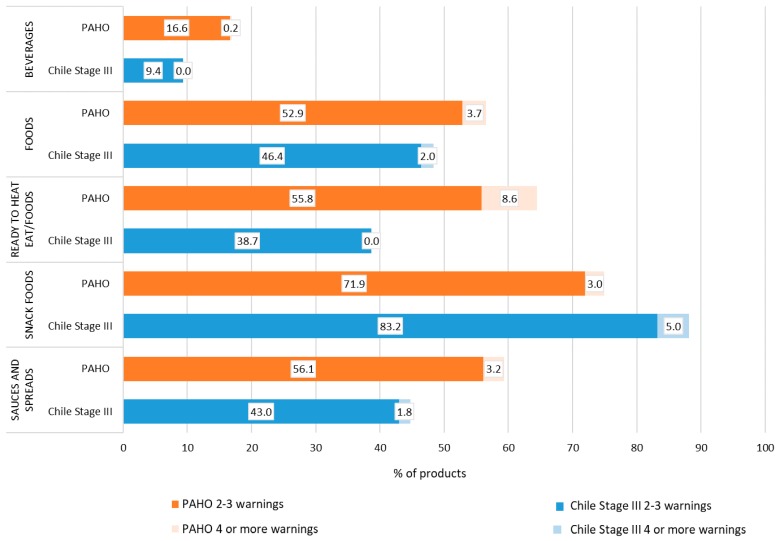
Proportion of beverage and food products with one, two or more, and four or more “excess nutrients of concern” under the Pan American Health Organization (PAHO) and Chile Stage III nutrient profiling systems.

Figure 3 shows the breakdown of the number of NOCs exceeding thresholds under the PAHO NPS and under the Chile Stage III NPS. Under the PAHO NPS, 66.1% of beverages have only one NOC in excess (Table S1 shows that this is primarily from excess sugar), with another 16.6% having a second or third NOC in excess (Table S1 suggests that this is explained by the presence of artificial sweeteners or excess sodium). Under the Chile Stage II NPS, 57.5% of beverages have only one NOC in excess (Table S2 shows that this is primarily from excess sugar), with another 9% having a second or third NOC in excess (Table S2 suggests that this is explained by excess energy).

Under the PAHO NPS, 33.6% of foods would have only one NOC in excess, 52.9% would have two or three NOCs in excess and less than 4% would have four or more NOCs in excess. Only 24% of snack foods exceed only one NOC threshold, while 71.9% exceed two to three and 3% exceed four or more NOC thresholds. Likewise, the majority of ‘ready to heat/eat foods’ (64.4%) and ‘sauces and spreads’ (59.3%) have two or more NOCs exceeding PAHO thresholds. This contrasts with the proportion of ‘fruits and vegetables’, ‘legumes’, ‘fish and seafood’, and ‘meat and eggs’ that had only one of the NOCs in excess of PAHO thresholds (Table S1).

Under the Chile Stage III NPS, 31.6% of foods would have only one NOC in excess, 46.4% would have two or three NOCs in excess and less than 2% would have four or more NOCs in excess. About 10% of ‘snack foods’ exceed only one nutrient threshold, while 83.2% exceed two to three and 5% exceed four or more nutrient thresholds. Unlike under PAHO, there is more of an even distribution of ‘ready to heat/eat foods’ and ‘sauces and spreads’ containing only one excess NOC vs. two or more excess NOCs when using the Chile Stage III NPS. Meanwhile, the majority of ‘fruits and vegetables’, ‘fish and seafood’, and ‘meat and eggs’ with any NOC in excess of Chile Stage III thresholds only had one NOC in excess (see Table S2).

Differences in the share of “excess” NOC products between the PAHO and the Chile Stage III NPSs were explored using pairwise correlation coefficients and tests of proportions, as shown in Table 2. The highest correlation was for beverages with any excess NOCs, but the lowest correlation was also for beverages with regards to the number of excess NOCs. Again, there is a significantly larger percentage of products in beverage and food categories considered to have excess NOCs under the PAHO NPS compared to the Chile Stage III NPS. This is especially the case for sodium and sugar in beverages, as well as sodium and sugar in foods.

### 3.2. Determining Sources of Differences across NPS

**Figure 4:**
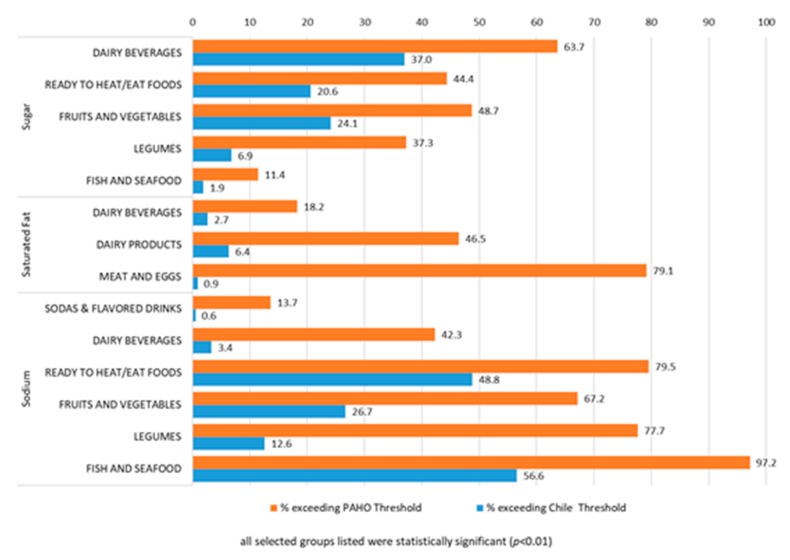
Significant differences in percentage of beverages and foods exceeding nutrient thresholds by each nutrient profiling system.

When comparing the three common NOCs considered in both NPSs (sugar, saturated fat, sodium), there are certain stark and statistically significant differences, with the PAHO NPS having a notably larger share of products exceeding each of these nutrients (Figure 4). Under the PAHO NPS, products exceed the sugar threshold when calories from sugar constitute ≥10% of energy (whereas, under the Chile NPS, it is based on sugar density of ≤5 g for beverages and ≥10 g for foods). This was the case for 64% of ‘dairy beverages’ (vs. 37% under Chile, *p =* 0.00), 44% of ‘ready to heat/eat foods’ (vs. 21% under Chile, *p =* 0.00), 49% of ‘fruits of vegetables’ (vs. 24% under Chile, *p =* 0.00), 37% of ‘legumes’ (vs. 7% under Chile, *p =* 0.00) and 11% of ‘fish and seafood’ (vs. 2% under Chile, *p =* 0.00).

Likewise, under the PAHO NPS, products exceed saturated fat thresholds when calories from saturated fat constitute ≥10% of energy (whereas, under the Chile NPS, it is based on a saturated fat density of ≥3 g for beverages and ≥4 g for foods). This was the case for 18% of ‘dairy beverages’ (vs. 3% under Chile, *p =* 0.00), 46% of ‘dairy products’ (vs. 6% under Chile, *p =* 0.00), and 79% of ‘meats and eggs’ (vs. 1% under Chile, *p =* 0.00).

Finally, for sodium, products exceed sodium thresholds when the ratio of sodium in mg to energy in kilocalories is greater than one under the PAHO NPS (whereas, under the Chile NPS, it is based on sodium density ≥100 mg for beverages and ≥400 mg for foods). Under PAHO, 14% of ‘sodas and flavored drinks’ (vs. 0.5%, *p =* 0.00), 42% of ‘dairy beverages’ (vs. 3%, *p =* 0.00), 80% of ‘ready to heat/eat foods’ (vs. 49%, *p =* 0.00), 67% of ‘fruits of vegetables’ (vs. 27%, *p =* 0.00), 78% of ‘legumes’ (vs. 13%, *p =* 0.00) and 97% of ‘fish and seafood’ (vs. 57%, *p =* 0.00) would exceed sodium thresholds compared to under the Chile NPS.

Next, the nutrient values among the subset of products that overlapped in both NPSs were examined, comparing products that exceeded NOC under PAHO but not under Chile, and products not exceeding any NOCs in both NPS. Table 3 shows the nutrient densities (in terms of per 100 mL for beverage and per 100 g for foods), percent of energy from key NOCs and presence of artificial sweeteners for these various subsets of products. Focusing on those with excess NOCs under PAHO but not Chile provides a better understanding of why there are differences in the findings across the two NPSs.

Among the 214 beverages that would not exceed any NOC under Chile but do under PAHO, the mean calories per 100 mL is significantly lower than those without excess NOCs under both NPSs (26.21 kcals vs. 48.78 kcals, *p =* 0.00). However, 56% of calories comes from sugar and 32% of calories comes from fat, the mean sodium:calorie density ratio is greater than one in this subset of beverages, and 46.3% of these products contain artificial sweeteners. Beverage products without excess NOCs under both systems have the lowest sugar density, the lowest share of products containing artificial sweeteners, lowest sodium density and ratio, and lowest total fat and saturated fat densities across the three subsets of beverages.

Among the 374 food products that would not exceed any NOCs under Chile but do under PAHO, both mean caloric density as well as sugar density are lower than those with no excess NOCs under either NPS. Moreover, compared to those with any excess NOCs under both NPSs, these products have a significantly lower sugar density (3.38 g vs. 19.84 g, *p =* 0.00), percent of calories from sugar (21% vs. 30%, *p =* 0.00), sodium density (263 mg vs. 1359 mg, *p =* 0.00), sodium ratio (3.42 vs. 6.66, *p =* 0.00), total fat density (3.42 g vs. 12.66 g, *p =* 0.00) and percent of calories from total fat (19% vs. 30%, *p =* 0.00). Food products without excess NOCs under both systems have the lowest percent of calories from sugar, sodium density and ratio, total fat density and percent of calories from total fat and saturated fat, but had the highest share of products containing artificial sweeteners.

## 4. Discussion

The primary goal was to compare what the potential application of two existing NPSs might mean, given food label data on the beverage and food products available for sale in Jamaica. In general, a larger percentage of beverages and foods exceed NOC thresholds under the PAHO NPS compared to the Chile Stage III NPS. Additionally, the combination of different NOCs included under each NPS and the reference units used (total energy for PAHO versus product volume or weight for Chile) can result in very different shares of products considered to have excess NOCs.

Given the data available from Jamaica, the Chile Stage III NPS appears to more parsimoniously categorize products as having excess NOCs compared to the PAHO NPS based on the following observations. Under PAHO, a significantly larger share of fruits, vegetables, legumes, fish and seafood, would be considered to have excess sugar or sodium. This reflects the pervasiveness of unhealthy manufacturing practices on changing food composition and could thereby thwart promotional efforts encouraging these foods that are already low in Jamaican diets. Additionally, the sodium ratio in the PAHO NPS may pose challenges for low or non-caloric beverages. Indeed, because the PAHO NPS uses energy/kilocalories as the reference unit (rather than product volume or weight), low calorie beverage and food products may be unduly penalized, as shown in Table 3. For beverages, those products with excess under PAHO but not Chile have lower caloric density and calories from sugar. Moreover, the use of energy/kilocalories as the reference unit may create perverse incentives for manufacturers to increase the energy density of their products to avoid select nutrients being considered in excess.

Additionally, the PAHO NPS includes more nutrients/ingredients of concern than the Chile NPS, including total fat and trans fat. To date, other countries that have passed or implemented regulations based on nutrient criteria have not included total fats. This is likely due to the current scientific evidence that shows that it is the type of fat, not total fat, that matters for health [23]. Trans fat in food products might be a nutrient of concern in Jamaica that deserves attention and thus may be considered as a component of an NPS for Jamaica. Therefore, should the PAHO NPS be considered or adapted for Jamaica, it is recommended that these changes be considered: (i) the sodium criteria be dropped for beverages or adjustments made in relation to sodium density; and, (ii) the total fat criteria be dropped in place of a criteria for energy density, which is considered only if a product contains any added sugar, sodium or fat.

In the absence of any formative food composition data, it is imperative to test how foods available in Jamaica perform using different NPSs. Other countries have compared how different NPSs might apply to the products available in their country, such as in Canada [24,25], Mexico [26] and Colombia [27]. The findings here on the share of products with excess NOCs under the PAHO compared to the Chile NPS are consistent with the studies done elsewhere; the PAHO NPS would identify a larger percentage of packaged products as having excess NOCs.

NPS can be useful tools for creating integrated food and nutrition policies, such as front-of-package labeling, marketing regulations, what products are permitted to be sold in government settings like public schools or hospitals, and fiscal policies. One primary use of NPS recently has been for front-of-package warning labels, such as in Chile, Peru and Uruguay [17,28,29]. Within the current setting, a stringent NPS would result in the majority of foods in Jamaica requiring multiple warning labels. However, there is a lack of evidence around whether, and to what extent, having one vs. more warning labels are interpreted differently by consumers and whether it would trivialize their relevance.

Jamaica faces a challenging decision regarding the choice of NPS, since a large proportion of beverage and food products exceed nutrient threshold parameters under both models. Careful selection of the appropriate NPS for use given a particular context and learning lessons from other countries that have applied various NPSs in policies is important in order to maximize the effectiveness of efforts to discourage unhealthy diets and provide a supportive food environment. There are arguments supporting the adoption of either the PAHO or Chile NPS. The Pan-American Health Organization has a long-standing program of technical cooperation with Jamaica and is instrumental in supporting government strategies for obesity prevention and reduction. The PAHO NPS is therefore expected to contribute to the final decisions establishing the nutrient threshold criteria and food label standards. As an alternative solution, the simpler Chilean NPS has succeeded with several policy iterations and appears to be readily accepted by consumers. The model continues to make remarkable achievements in enforcing labelling policies, restricting the marketing of unhealthy food to children, unhealthy beverage or food taxation, and driving healthier food reformulations.

The food labels of prepackaged foods and NPS are but two methods to inform consumers about healthy/healthier or unhealthy/unhealthier foods. Consumers should also be educated about the dangers of other frequently consumed unhealthy meals that would naturally be excluded from NPS categorization. Many traditional meals and beverages (e.g., meat stews, homemade teas and fruit drinks), may include excess amounts of saturated fats, sodium and added sugars, depending on methods of preparation. Also, more consumers are dependent on meals prepared outside the homes. Promoting healthier methods of preparation and implementing monitoring systems (e.g., menu nutrient declarations) for commercially prepared meals are suggested options for future research.

The limitations of this study include the inability to determine the representativeness of products included in the final sample and, thus, each product was treated equally rather than being weighted by market share. Jamaica is currently conducting a revision of its food labelling system in keeping with the Government’s remit to curb the availability and consumption of unhealthy foods. These data were collected in 2018, reflecting products currently available in the marketplace in Jamaica. Though nutrition labels are not yet mandatory (but ingredient lists are), the accuracy of nutrient information may be questionable; but this highlights the need for mandatory back-of-package regulations that include these key nutrients of concern. However, given that the objective of this paper was to compare two NPS, there is no reason to believe this will bias the findings.

## 5. Conclusions

This is the first study in the Caribbean region that applies two existing NPSs to a large sample of packaged beverage and food products sold in Jamaica. Given the array of local and imported products in Jamaica, the majority of the packaged food supply exceeded thresholds for nutrients of concern using either NPS. Using the PAHO NPS would result in a larger percentage of products being categorized as having excess nutrients of concern. The combination of different nutrients included under each NPS, and the reference units used, partially explain the different results. However, regardless of the NPS used these results support advocating for accurate food labeling systems, healthier food and beverage reformulations, and for manufacturers to improve food processing practices. Careful consideration is needed on whether and how these NPSs should be used or adapted to guide policies to improve food offerings and choices in Jamaica.

## Figures and Tables

**Table 1:** Brief description of the Pan American Health Organization and the Chilean Stage III nutrient profiling systems.

	Pan American Health Organization Nutrient Profiling System [13] (2016)	Chile Stage III [17] (Implemented in 2019)	
		Liquids	Solids
Products covered by nutrient thresholds	Any item that is processed, identified by the presence of added sweetener, salt, saturated fat, in ingredients list	Any packaged product with added ingredients representing added sugar, saturated fat, sodium/salt.	
Exclusion criteria	Culinary ingredients or single ingredient items	Infant formula, medical use and weight control products, fast foods, bulk/unpackaged foods, prepared meals.	
Sugar	Added sugar and free sugar is ≥10% of calories	Total sugar ≥5 g/100 mL	Total sugar ≥10 g/100 g
Saturated Fat	≥10% of calories	≥3 g/100 mL	≥4 g/100 g
Sodium	≥1 mg sodium: 1 kcal	≥100 mg/100 mL	≥400 mg/100 g
Calories	N/A; used as base for other nutrients	≥70 kcal/100 mL	≥275 kcal/100 g
Total Fat	≥30% of calories	Not applicable (not a nutrient of concern considered)	
Trans Fat	≥1% of calories	Not applicable (not a nutrient of concern considered)	
Low-caloric sweeteners	Presence	N/A, but Chile regulations require amounts of low-caloric sweeteners to listed on Nutrition Facts Panel	

**Table 2:** Pairwise correlation coefficients and differences in percent of products with excess nutrients of concern between PAHO NPS and Chile Stage III NPS.

	Pairwise Correlation Coefficients		Difference in % with Excess Nutrient (PAHO %–Chile %)					
	Any Nutrient in Excess	Number of Nutrients in Excess	High in Sugar	High in Saturated Fat	High in Sodium	Only One Nutrient in Excess	Two or Three Nutrients in Excess	Four or More Nutrients in Excess
Beverages	0.638	0.309	11.3 **	2.3 **	15.1 **	16.2 **	7.3 **	0.2
Foods	0.592	0.539	7.8 **	5.9 **	9.1 **	9.9 **	6.1 **	1.7 **
All	0.614	0.56	8.8 **	4.9 **	10.8 **	11.6 **	6.5 **	1.3 **

Notes: Pairwise (or matched) correlations conducted using -pwcorr- and tests of proportion conducted using -prtest- in Stata 15. ** denotes *p <* 0.01. * denotes *p <* 0.05.

**Table 3:** Nutrient values and among various subset of sample under PAHO and Chile Stage III NPS.

	Any Excess NOCs under PAHO and Chile	Excess NOCs under PAHO but Not Chile	No Excess NOCs under Both
**Beverages**	*N* = 878	*N* = 214	*N* = 223
Calories kcal per 100 mL (mean, SE)	61.74 (1.99)	26.21 (1.82)	48.78 (1.41)
Sugar g per 100 mL (mean, SE)	23.57 (1.38)	30.34 (2.59)	11.38 (0.74)
Calories from Sugar (%)	89%	56%	81.9%
Contains artificial sweetener (%)	17%	46.3%	2.2%
Total fat g per 100 mL (mean, SE)	0.61 (0.10)	1.05 (0.19)	0.36 (0.12)
Calories from Total Fat (%)	6%	31.6%	7.4%
Saturated fat g per 100 mL (mean, SE)	0.19 (0.04)	0.39 (0.10)	0.16 (0.07)
Calories from Saturated Fat (%)	1%	8.7%	1.4%
Sodium mg per 100 mL (mean, SE)	28.00 (2.50)	33.29 (6.85)	12.06 (1.11)
Sodium: kcal ratio (mean, SE)	0.59 (0.06)	2.25 (0.31)	0.28 (0.03)
**Foods**	*N* = 2709	*N* = 374	*N* = 310
Calories kcal per 100 g (mean, SE)	304.29 (3.52)	102.72 (4.48)	212.33 (8.94)
Sugar g per 100 g (mean, SE)	19.84 (0.41)	3.38 (0.16)	5.62 (0.87)
Calories from Sugar (%)	30%	21.1%	11.9%
Contains artificial sweetener (%)	3%	9.1%	16.1%
Total fat g per 100 g (mean, SE)	12.66 (0.31)	3.42 (0.36)	2.42 (0.34)
Calories from Total Fat (%)	30%	18.8%	9.2%
Saturated fat g per 100 g (mean, SE)	4.31 (0.11)	0.86 (0.14)	0.87 (0.21)
Calories from Saturated Fat (%)	10%	4.3%	3.6%
Sodium mg per 100 g (mean, SE)	1358.39 (66.59)	263.48 (9.81)	247.87 (84.36)
Sodium: kcal ratio (mean, SE)	6.66 (0.42)	3.91 (0.17)	0.58 (0.16)

Notes: Only one beverage product and 30 food products (<0.7% of overall sample) have any excess nutrients under Chile but not PAHO. NOC = nutrients of concern.

## Supplementary Materials

The following are available online at https://www.mdpi.com/2304-8158/9/1/65/s1, Figure S1: Flowchart used for applying the Pan American Health Organization nutrient profiling system, Figure S2: Flowchart used for applying the Chile Stage III nutrient profiling system, Table S1: Proportion of beverage/food products with various types and numbers “excess nutrients of concern” under the Pan American Health Organization nutrient profiling system, Table S2: Proportion of beverage/food products with various types and numbers “excess nutrients of concern” under the Chile Stage III nutrient profiling system.
