# Supplementary material for: Applying Nutrient Profiling Systems to Packaged Foods and Drinks Sold in Jamaica
Source: Foods. 2020 Jan 8;9(1):65. doi: 10.3390/foods9010065 (PMC7022911; doi:10.3390/foods9010065)
Supplement: Supplementary file 1 [file foods-09-00065-s001.pdf]

Figure S1: Algorithm used to evaluate Jamaican products for PAHO nutrient profiling system

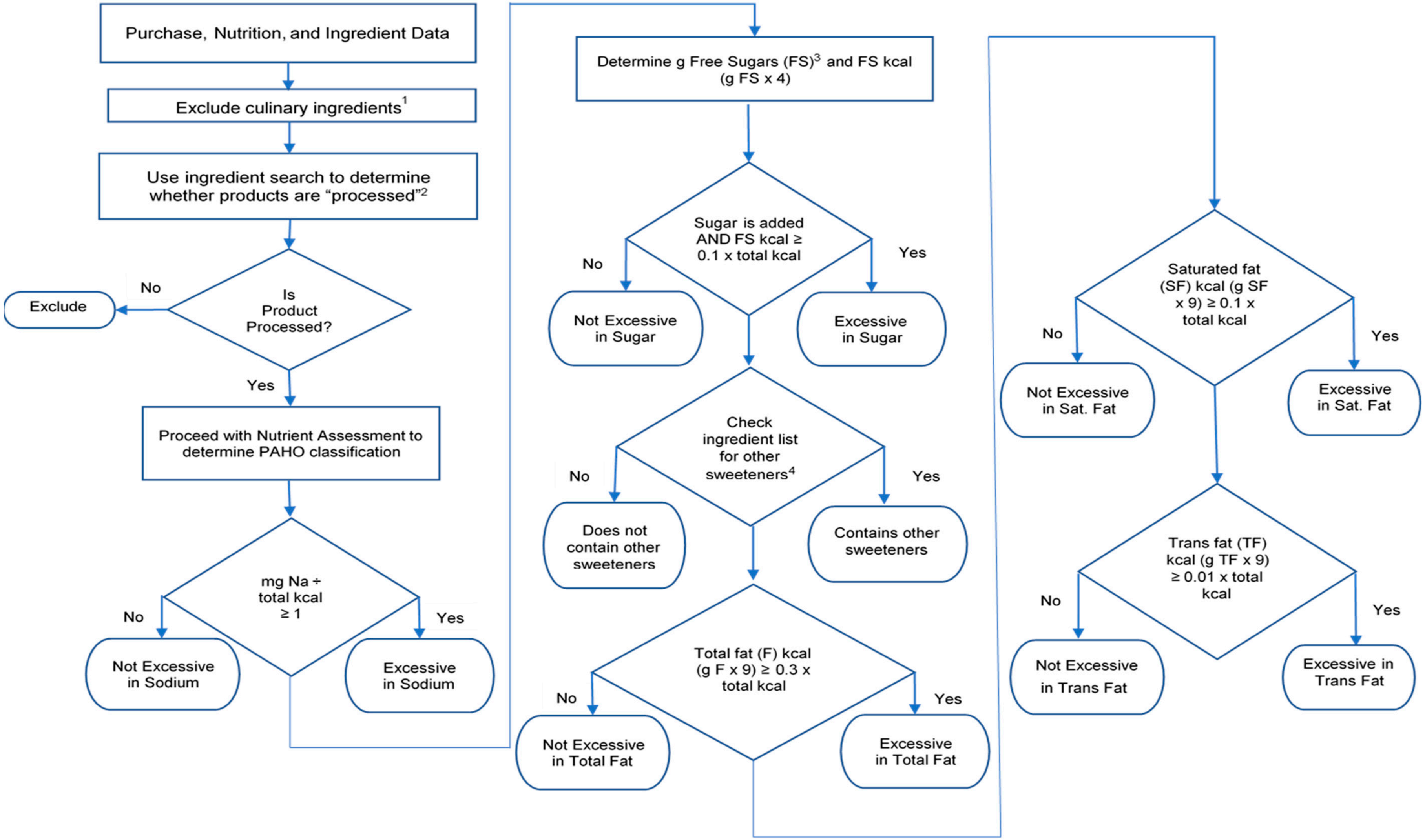

Notes for PAHO nutrient profile

1. Exclude Culinary Ingredients

**Examples of Products Excluded as Culinary Ingredients**
Oils
Butter
Caloric Sweeteners
Low Calorie Sweeteners
Other Sweeteners

2. Search product ingredients using lists of ingredients representing added fats, sugars, artificial sweeteners, and salt. Lists of ingredients developed by Global Food Research Program based on various food databases, food manufacturing ingredients, policies, and regulations. Presence of any of these components indicates product is processed. Food items containing no additional ingredients are excluded.

3. Steps for estimating Free Sugars (FS) [only for processed products]:
   a. If product contains 0g Total Sugar, set FS = 0g
   b. If product contains >0g Total Sugar, Global Food Research Program nutritionists review categories & products to confirm or reassign FS value
      i. Categories requiring nutritionist review: (may contain fruit/milk in the ingredient list, necessitating change in FS value)

Coffee	Desserts
Tea	Candy - Chocolate
Dairy drinks	Candy - Non Chocolate
Meal Replacement/Supplements	Other Sweets
Syrups	Ready-to-eat baby foods
Other Beverages	Other Dairy
Sweet Baked Goods	Dried fruits
Other Bakery Products	Other Fruits and Vegetables
Breakfast cereals	Sweet Sauces & Spreads
Granola bars	Nuts & Seeds
Other Cereal & Grain Products	Plantain/Banana Chips
Ice creams	Non-dairy creams
Sorbet	Other Sweeteners

ii. Note – PAHO Rules for determining FS:

Method for estimating free sugars based on the amount of total sugars declared on food/beverage product packaging		
**If the manufacturer declares…**	**Then estimated free sugars equal…**	**Examples of products**
0g of total sugars	0g	Canned fish
added sugars	declared added sugars	Any product that declares added sugars
total sugars, and the product is part of a group of foods with no or a minimal amount of naturally occurring sugars	declared total sugars	Regular soft drinks, sport drinks, sweet biscuits, breakfast cereals, chocolate, and savory and sweet biscuits
total sugars and the product is yogurt or milk, with sugars in the list of ingredients	50% of declared total sugars	Flavored milk or yogurt
total sugars, and the product is a processed fruit item with sugars in the list of ingredients	50% of declared total sugars	Fruit in syrup
total sugars, and the product has milk or fruit in the list of ingredients	75% of declared total sugars	Cereal bar with fruit

4. Search product ingredients using lists of ingredients representing added fats, sugars, artificial sweeteners, and salt. Lists of ingredients developed by Global Food Research Program based on various food databases, food manufacturing ingredients, policies, and regulations.

Figure S2: Algorithm used to evaluate Jamaican products for Chile Stage III nutrient profiling system

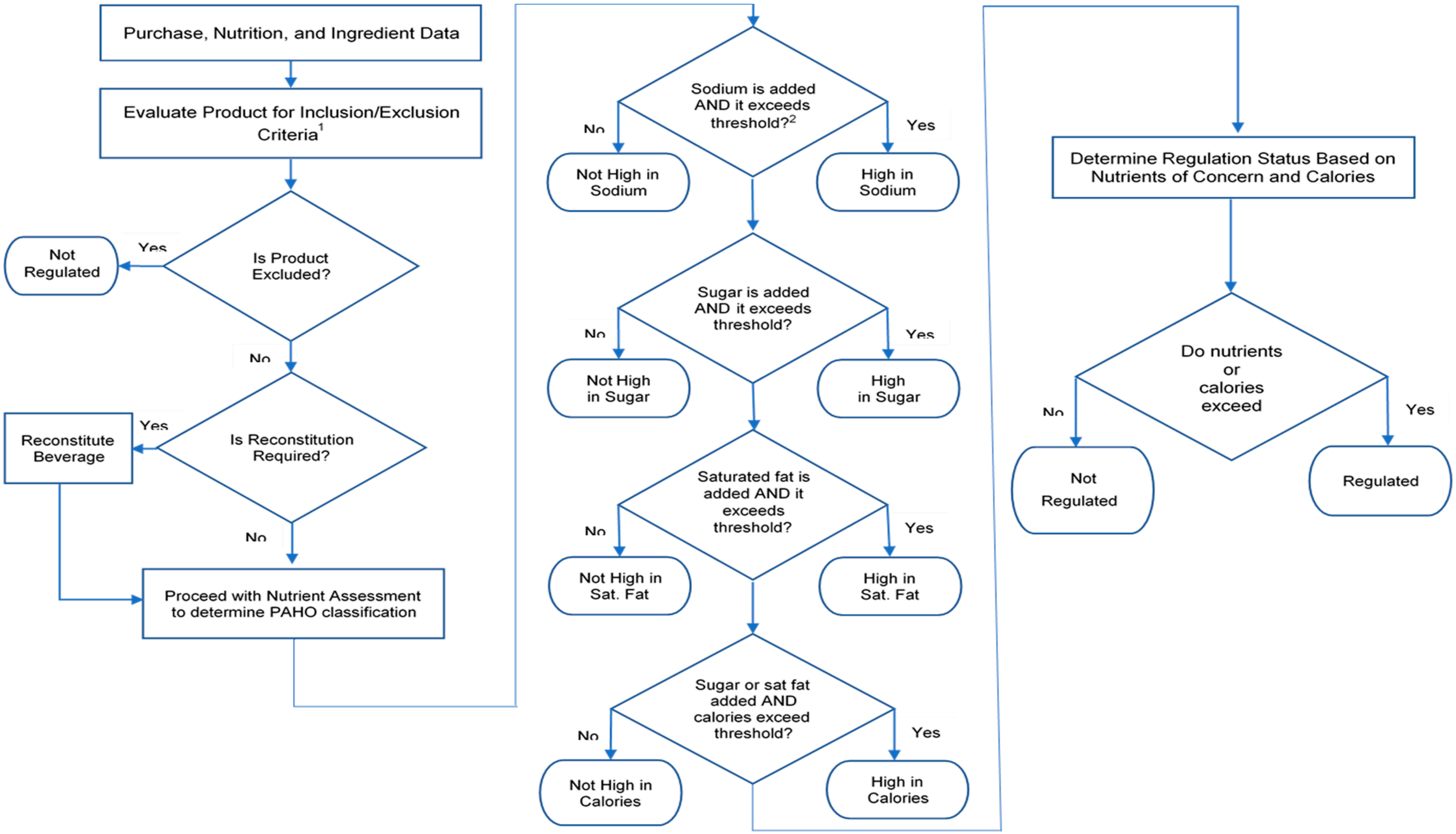

Figure S2: Algorithm used to evaluate Jamaican products for Chile Stage III nutrient profiling system

NOTES

1. Exclusion criteria in Chile regulations
    i. No added ingredients of concern (Search product ingredients using lists of ingredients representing added sugar, saturated fat, sodium/salt, etc. Lists of ingredients developed by Global Food Research Program based on various food databases, food manufacturing ingredients, policies, and regulations. Food items containing no ingredients of concern are excluded.)
    ii. Infant formula – ALL formulas are excluded from labelling and marketing regulations at this time due to gap in the Reglamento Sanitario De Los Alimentos (statement refers to formulas *beyond 4-6 months* without an upper age limit); This is to be corrected with updated regulations once Ley 20869 is actualized
        1. NAN formula products – none are regulated at this time (1/2018)
        2. Nido etapa formula products – none are regulated at this time (1/2018)
    iii. Fast foods, bulk or unpackaged food items, prepared meals even if packaged at time of sale
        1. NFP labels not currently on these items, thus labelling regulations do not currently apply
        2. These products are subject to advertising/marketing regulations if they contain added ingredients of concern that exceed thresholds
    iv. Commercial preparations of infant foods (coladas, picadas), unless they contain added sugar
    v. Processed cereal based baby food, unless they contain added sugar
    vi. Medical use products
    vii. Weight control products

2. Note that, for Jamaica, the assessment is based on the Chile-2019 nutrient thresholds.

Chile - 2019 Nutrient Thresholds	Food	Beverage
**Energy (kcal/100g)**	275	70
**Total sugar (g/100g or ml)**	10	5
**Sodium (mg/100g or ml)**	400	100
**Saturated fat (g/100g or ml)**	4	3

Table S1: Proportion of food/beverage products with various types and numbers "excess nutrients of concern" under the Pan American Health Organization nutrient profiling system

	N	% "processed"	% excess sugar	% contains artificial sweetener	% excess sodium	% excess saturated fat	% excess total fat	% excess trans fat	Any excess nutrients	2-3 excess nutrients	4 or more excess nutrients
**Beverages**	1316	84.4	76.0	19.5	17.0	2.8	8.3	0.0	83.0	16.6	0.2
Sodas and flavored waters	894	96.8	91.3	22.5	13.7	0.8	1.4	0.0	95.6	10.4	0.0
Dairy drinks	149	93.3	63.7	6.7	42.3	18.2	44.3	0.0	87.9	55.0	2.0
100% juice and coconut waters	171	5.3	1.8	0.0	3.5	0.0	0.0	0.0	5.3	0.0	0.0
Other beverages	102	96.1	84.3	45.1	30.4	2.0	29.4	0.0	95.1	43.1	0.0
**Foods**	3423	93.6	50.4	4.5	56.9	29.3	41.8	2.3	90.1	52.8	3.7
Bread and bakery products	549	100.0	60.7	3.8	52.0	32.1	45.5	2.7	94.4	56.8	3.8
Candy and desserts	376	99.7	86.4	19.1	3.0	50.3	56.9	5.9	88.0	52.4	6.4
Snack foods	363	99.7	14.1	1.9	59.8	46.0	88.4	1.1	98.9	71.9	3.0
Ready to heat/eat foods	163	95.7	44.4	3.7	79.5	36.6	41.7	1.9	94.5	55.8	8.6
Cereal and grain products	242	66.1	47.5	2.1	37.2	1.7	6.6	0.0	61.2	30.2	0.0
Dairy products	299	92.6	45.8	7.0	46.8	46.5	49.8	7.7	92.6	44.1	6.4
Fruits and vegetables	195	90.8	48.7	3.1	67.2	3.7	8.4	0.0	89.7	34.9	0.0
Legumes	103	79.6	37.3	0.0	77.7	1.9	1.9	0.0	77.7	38.8	0.0
Meat and eggs	108	95.4	3.1	0.0	91.7	79.1	85.2	11.9	95.4	79.6	5.6
Fish and seafood	106	100.0	11.4	0.9	97.2	49.0	72.6	0.0	99.1	74.5	3.8
Meat substitutes and dairy substitutes	31	80.7	10.7	0.0	58.1	12.9	71.0	3.2	80.6	58.1	0.0
Sauces and spreads	745	95.0	64.0	2.1	70.6	16.1	25.9	0.3	92.8	56.1	3.2
Dry spices and seasonings	143	86.7	37.0	0.0	80.4	2.8	4.9	0.0	81.8	23.8	1.4
**All Beverages and Foods**	4739	91.1	57.6	8.7	45.9	21.9	32.6	1.6	88.1	42.8	2.7

Notes: See Appendix S1 for details on the PAHO Nutrient Profiling System algorithm and definitions

Table S2: Proportion of food/beverage products with various types and numbers "excess nutrients of concern" under the Chile Stage III nutrient profiling system

	N	% containing added ingredients of concern	% excess sugar	% excess sodium	% excess saturated fat	% excess calories	Any excess nutrients	2-3 excess nutrients	4 or more excess nutrients
**Beverages**	64.7	81.5	1.8	0.5	9.8	66.8	9.3	0.0	64.7
Sodas and flavored waters	81.0	92.5	0.6	0.0	6.6	81.5	6.6	0.0	81.0
Dairy drinks	37.0	93.3	3.4	2.7	11.4	39.6	12.1	0.0	37.0
100% juice and coconut waters	1.8	5.3	2.9	0.0	0.0	4.7	0.0	0.0	1.8
Other beverages	66.7	96.1	8.8	2.0	52.0	81.4	45.1	0.0	66.7
**Foods**	42.4	92.3	47.4	23.3	44.7	80.0	46.4	2.0	42.4
Bread and bakery products	58.5	100.0	48.7	37.8	82.3	92.9	73.2	5.6	58.5
Candy and desserts	86.1	89.4	2.1	52.7	63.3	86.7	75.3	0.5	86.1
Snack foods	16.3	99.7	68.6	59.2	96.1	98.1	83.2	5.0	16.3
Ready to heat/eat foods	20.6	95.7	48.8	22.1	36.2	65.0	38.7	0.0	20.6
Cereal and grain products	47.1	66.1	32.2	6.2	57.9	62.4	54.5	0.8	47.1
Dairy products	39.5	91.3	42.8	6.4	9.0	82.9	10.4	0.0	39.5
Fruits and vegetables	24.1	90.8	26.7	0.5	3.6	46.7	6.7	0.0	24.1
Legumes	6.9	79.6	12.6	0.0	1.9	18.4	1.9	0.0	6.9
Meat and eggs	0.0	95.4	82.4	0.9	8.3	83.3	7.4	0.0	0.0
Fish and seafood	1.9	100.0	56.6	5.7	1.9	63.2	2.8	0.0	1.9
Meat substitutes and dairy substitutes	10.7	80.6	51.6	9.7	25.8	74.2	12.9	0.0	10.7
Sauces and spreads	49.6	94.8	62.5	12.7	29.4	85.2	43.0	1.7	49.6
Dry spices and seasonings	28.3	86.7	79.7	0.7	13.3	81.8	18.2	0.7	28.3
**All Beverages and Foods**	4739	89.3	48.7	34.7	16.9	35.0	76.3	36.1	1.4

Notes: See Appendix S2 for details on the Chile Stage III Nutrient Profiling System algorithm and definitions
